# New Perspective on Natural Plant Protein-Based Nanocarriers for Bioactive Ingredients Delivery

**DOI:** 10.3390/foods11121701

**Published:** 2022-06-09

**Authors:** Chaoting Wen, Jixian Zhang, Haihui Zhang, Yuqing Duan

**Affiliations:** 1 College of Food Science and Engineering, Yangzhou University, Yangzhou 225127, China; chaoting@yzu.edu.cn; 2 School of Food and Biological Engineering, Jiangsu University, Zhenjiang 212013, China; zhanghh@ujs.edu.cn

**Keywords:** plant protein, nanocarriers, preparation method, bioactive substances, bioavailability

## Abstract

The health effects of bioactive substances in the human body are affected by several factors, including food processing conditions, storage conditions, light and heat, among others. These factors greatly limit the stability and bioavailability of bioactive substances. These problems can be solved by a novel protein-based nanocarrier technology, which has the excellent potential to enhance solubility, bioavailability, and the controlled release of bioactive substances. In addition, plant protein has the advantages of economy, environmental protection, and high nutrition compared to animal protein. In this review, the preparation, characterization, and application of plant protein-based nanocarriers are summarized. The research deficiency and future prospects of plant protein nanocarriers are emphasized.

## 1. Introduction

In recent decades, there has been increasing research on natural bioactive compounds (polyphenols, vitamins, carotenoids, etc.,) due to their anti-oxidative, anti-cancer, mediating cardiovascular and neurodegenerative activities, among others [1,2]. However, these active compounds are generally unstable under light, heat, oxygen, and certain conditions [3]. In addition, some active compounds have low bioavailability due to low water solubility and gastrointestinal instability, which limits their application in functional foods, nutritional supplements, and pharmaceutical products [4].

**Figure 1:**
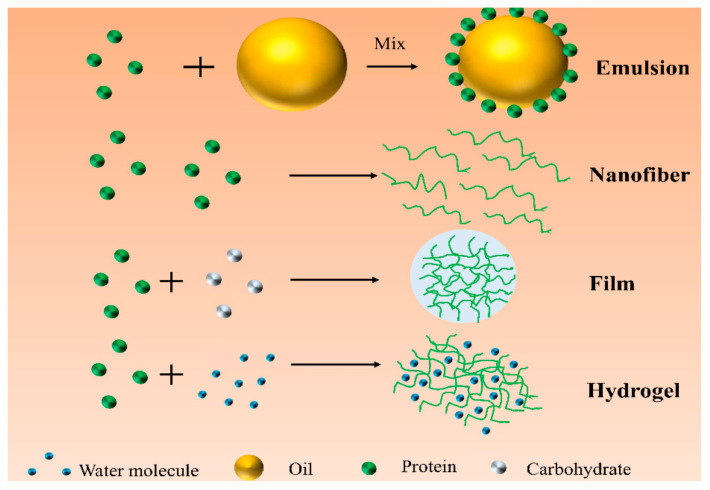
Schematic diagram of different types of protein-based nano-carriers.

Nanocarriers can protect active substances from adverse external factors and gastrointestinal degradation, and they can improve the solubility, functional properties, and biological activity of active substances [5]. Proteins are used to prepare nanocarriers due to their high biocompatibility, amphiphilicity, easy degradability, digestibility, edibleness, and nontoxicity [6]. Proteins are not only prepared into different types of nanocarriers to meet different application requirements due to the variability of their structural design, but also it can protect bioactive substances from oxidative degradation by scavenging free radicals [7]. In addition, proteins have multiple functional groups and are easy to interact with other biopolymers to form nanocarriers, which can significantly improve the solubility, biocompatibility, and bioavailability of bioactive substances [8]. Thus, it can be seen that protein nanocarriers have great potential and application prospects in the delivery of bioactive substances. As shown in Table 1, animal-derived protein-based nanocarriers have been widely used to deliver active substances due to their abundant sources, high activity, good biocompatibility, and nutritional value, among others [9]. However, animal protein-based nanocarriers have problems such as a high proportion of cholesterol, high price, and limited vegan consumers, among others [10]. Various plant protein-based nanocarriers can be used to replace animal-based nanocarriers due to their advantages of sustainability, low price, high targeting, and environmental protection, among others [11]. The main raw materials of plant-based nanocarriers include zein [12], soy protein [13], pea protein [14], and potato protein [15], among others. Plant protein-based nanocarriers mainly include nanofibers, emulsion, hydrogels, and films, among others [16] (Figure 1).

Protein nanoparticles can be used to prepare stabilizers for emulsions. Soy protein-resveratrol could accumulate at the water–in–oil interface, which could significantly increase the oxidative stability of the emulsion and avoid the formation of lipid hydroperoxides [17]. The zein–chitosan composite particles were prepared by wang et al., who used anti-solvent technology encapsulated curcumin to form an emulsion with strong antioxidant capacity [18]. Moreover, emulsion systems were prepared from vegetable protein-based particles, which also could be used as carriers for the delivery of biologically active substances. Steviol et al. found that glycoside-soy protein isolate emulsion could significantly enhance the delivery efficiency of resveratrol and improve its oxidative stability [17]. Zein and soy protein are widely used to develop nanofibers, which are mainly for delivery and controlled release applications. Many researchers had used electrospinning technology to produce zein nanofibers, which had successfully encapsulated and delivered bioactive substances such as gallic acid [19], and β-carotene [20], among others. Soy protein isolate-poly (ethylene oxide)-poly (lactic acid) nanofibers could effectively deliver allyl isothiocyanate to exert strong antibacterial activities [21]. In addition, soy protein isolate-poly (ethylene oxide) could also encapsulate the anthocyanin-rich red raspberry extract, which makes it highly resistant to staphylococcus [22]. Protein-based nanofibers had promising application prospects for the development of packaging materials for food delivery. Plant protein-based films are gaining increasing interest in food packaging [23]. Additionally, vegetable protein-based films offer good potential for the delivery and controlled release of lipophilic actives. Zein is also capable of forming films that can carry antimicrobials and antioxidants such as lysozyme [24], and various phenolic compounds [25], among others. The zein composite film was developed by Mastromatteo et al., who reported this film could retard the release rate of thymol. Cinnamaldehyde-crosslinked gliadin films also had been used as a carrier system for the release of lysozyme, mainly due to the ability of the protein’s network to control the rate of thymol release [26]. Hydrogels can regulate the release of active substances in the gastrointestinal tract by changing their microstructure [27]. Compared with synthetic polymer-based hydrogels, vegetable protein-based hydrogels had the advantage of biocompatibility and biodegradability, among others [28]. It was worth noting that plant protein-based hydrogels had good pH responsiveness due to the presence of a large number of acidic and basic groups in their polypeptide chains, thereby enabling effective delivery of active substances in the gastrointestinal tract [29]. Hydrogels were prepared from glycinin and dextran sulfate, which exhibited sustained drug release capabilities in simulated gastrointestinal digestion [30]. In addition, Scholten et al. prepared thermally responsive zein hydrogels that can effectively deliver hydrophobically active substances and avoid degradation by proteases [31]. However, choosing the ideal carrier for different biologically active compounds is a great challenge.

In this review, the latest research progress on plant protein-based nanocarriers to deliver bioactive substances were reviewed. Some advantages and disadvantages of the different preparation methods were described. In addition, recent applications of different types of plant protein-based carrier systems for the stabilization, protection, and delivery of active substances were discussed. Finally, the current deficiencies and prospects of plant-based nanocarriers for the delivery of active substances were also proposed.

## 2. Preparation and Affect Factors of Protein-Based Nanocarriers

**Figure 2:**
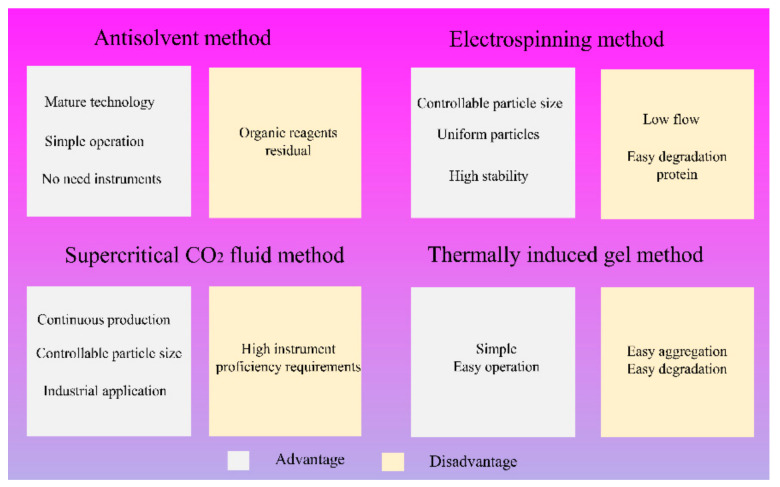
Schematic diagram of advantages and disadvantages of different methods for preparing protein-based nanocarriers.

Currently, the preparation methods of protein-based nanocarriers mainly include liquid–liquid dispersion/anti-solvent method, electrospinning, supercritical technology, and thermally induced gel method, among others. Marty et al. proposed the anti-solvent method in the 1978 year. The principle of this method is based on the addition of anti-solvent factors such as natural salts or ethanol into the protein solution, which can change the tertiary structure of the protein and promote the formation of protein aggregates [32]. The preparation of zein based nanoparticles by liquid–liquid dispersion has simple operation and good oxidation stability [33]. Patel et al. synthesized zein-curcumin colloidal particles by the antisolvent precipitation method. The entrapment efficiency and curcumin loading rate of the zein colloidal particles were 71.1–86.8% and 1.6–4.1%, respectively [34]. Zou et al. prepared zein-procyanidins nanoparticles by liquid–liquid dispersion method. The encapsulation efficiency of the zein–procyanidins nanoparticles was 86% and the shape was spherical. In addition, the infrared spectra showed that zein and procyanidins were connected by hydrogen bonds and hydrophobic interaction [35]. As shown in Figure 2, the liquid–liquid dispersion/antisolvent method has the advantages of mature technology, simple operation, and no need for instruments, but it needs organic reagents such as ethanol [11].

With the development of nanotechnology, electrospinning, as a facile and effective new processing technology to produce nanofibers, plays an important role in the delivery of active substances, food processing, and pharmacy, among others [36]. Electrospray spray has the advantages of controllable particle size, uniform particles, and high carrier stability, while it also has the disadvantages of low flow and easy degradation of the protein. Aytac et al. used electrospinning technology to encapsulate quercetin in a zein nanofiber matrix, which could significantly improve the water solubility, stability, and antioxidant activity of quercetin [37]. Dry nanoparticles can be obtained directly by electrospray, but special instruments are needed [6]. The supercritical CO_2_ fluid method can realize continuous production and prepare monodisperse nanoparticles with controllable particle size, which has a good prospect of industrial application [38]. However, this method requires operators to be familiar with the instrument. Compared with the other methods, the thermally induced gel method is simpler and easier to operate. Prolonged heat induction may lead to easy aggregation and degradation of proteins. Thermally induced gelation is a sequential process involving the opening of protein molecular structures and protein–protein interactions (e.g., hydrophobic interactions, electrostatic interactions, hydrogen bond, and disulfide bond formation, etc.) [39]. Chen et al. formed stable spherical nanogels by heating soybean protein dispersions at 95 °C, pH 5.9, and the internal structure of the nanogels was mainly stabilized by disulfide cross-linked networks and hydrophobic interactions [40]. Therefore, appropriate preparation methods can be selected according to different purposes in the experiment. More new-type preparation technologies also need to be further developed.

The structure can greatly affect the stability of nanocarriers in the food system during the process of digestion and absorption. It is very important to use a variety of analytical methods to characterize the molecular structure of nanocarriers in multiple dimensions. The characteristic indexes of nanocarriers mainly include particle size, zeta-potential, morphology, composition, physical state, mechanical characteristics, etc. Therefore, it is important to utilize effective methods for monitoring structural changes induced by the preparation method. These technologies mainly include particle size analyzer, laser confocal microscope, scanning electron microscope, atomic force microscope, transmission electron microscope, differential thermal scanning, Fourier transform infrared (FTIR), and X-ray diffraction, among others [41]. These techniques are particularly useful for detecting the structural transformation of plant protein-based nanocarriers.

**Figure 3:**
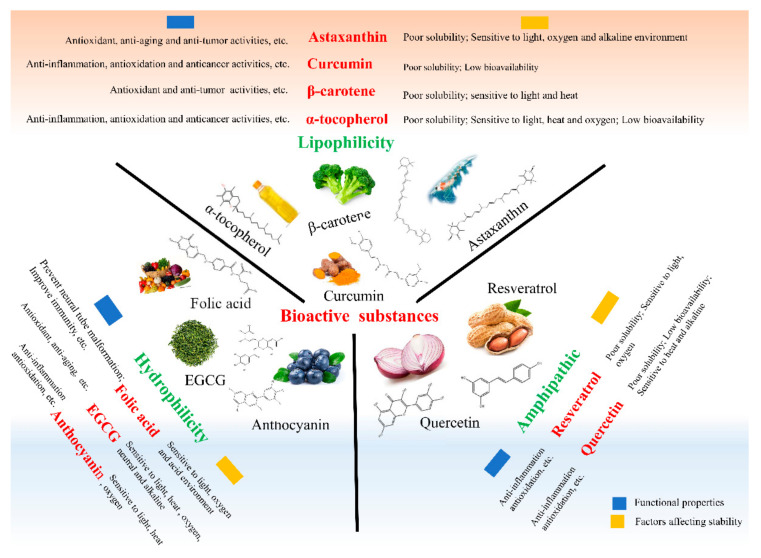
Schematic diagram of factors affecting the stability of bioactive substances.

As shown in Figure 3, the sources, biological activities, and application-limiting factors of different bioactive substances were listed. According to the different solubility of bioactive substances in the water and oil phase, they can be divided into three categories, namely lipophilic bioactive substances with good fat solubility, hydrophilic bioactive substances with good solubility in the water phase, and amphipathic bioactive substances [42]. Plant protein-based nanocarriers need to be developed according to specific characteristics of bioactive substances.

Bioactive substances are mostly sensitive to environmental factors, including light, oxygen, temperature, and pH during processing and storage. Lipophilic and amphiphilic bioactive substances are poorly compatible with aqueous products, which limits the absorption characteristics and bioavailability [43]. These factors affect the development and application of bioactive substances in functional products. Plant protein has high nutritional value and is widely used as an effective carrier for embedding bioactive substances to improve its photo, thermal stability, and prolong the shelf life [8].

## 3. Application of Bioactive Substances Loaded on Plant Protein-Based Nanocarriers

Nanocarriers are widely used to deliver active substances, mainly due to their ability to pass through the mucosa and higher bioavailability [44]. Plant protein-based nanocarriers are a new and ideal method to deliver active substances. Compared with animal protein nanocarriers, plant protein nanocarriers have the advantages of more stability, low price, and strong drug release ability [41]. In addition, plant protein nanocarriers have a high ability to load active substances, which is mainly due to their multiple binding sites and amphiphilic structure [11]. Protein nanocarriers bind to active substances mainly through hydrogen bonds, hydrophobic interactions, electrostatic attraction, and covalent bonds [11]. 

### 3.1. Zein-Based Nanocarriers

Zein has important application value in the food processing and active delivery field [45]. Zein is an amphiphilic molecule with self-assembly ability, which can form corresponding mesoporous structures in different solvents and effectively embedding hydrophobic active substances [46]. As shown in Table 2, Luo et al. prepared zein-based nanoparticles coated with carboxymethyl chitosan by the liquid–liquid dispersion method. The nanoparticles could significantly improve the encapsulation efficiency (87.9%), gastrointestinal stability, and optical stability of vitamin D3 (VD3). In addition, the nanoparticles were spherical with a particle size of 86–200 nm [47]. The low water solubility of tangeretin seriously limits its application in hydrophilic beverages and foods. Chen et al. used zein, β-lactoglobulin-coated tangeretin to improve its solubility. In addition, zein-tangeretin-β-Lactoglobulin nanoparticles had good stability in the condition of low concentration of salt and far away from the isoelectric point of β-Lactoglobulin protein [48]. Epigallocatechin gallate (EGCG) has been widely concerned by researchers because of its delaying fat digestion, antioxidation, and anticancer activities. However, EGCG has low stability during storage, processing, and digestion. The digestibility and encapsulation efficiency of EGCG could be significantly improved by zein-based colloidal particles embedding EGCG by using the antisolvent precipitation method [49]. Hu et al. reported that hollow zein–tannic acid nanoparticles were obtained by cross-linking. The particle size, PDI and zeta potential of hollow zein–tannic acid nanoparticles were 87.93 nm, 0.105, and −39.70 mV, respectively. Compared with solid zein–tannic acid nanoparticles, hollow zein–tannic acid nanoparticles had a higher potential for delivery of curcumin and encapsulation efficiency [50]. Caco-2 cell adhesion experiment in vitro showed that curcumin in zein–curcumin colloidal particles retained a longer time than free curcumin, with a value of 150 min [34]. The electrospraying technique was used to prepare zein nanoparticles encapsulated in green tea catechin by Bhushani et al. The results showed that the concentration of zein solution was 5% (*w*/*w*), and the diameter of the nanoparticles was 157 nm and showed a spherical shape. The zein nanoparticles could significantly improve the stability of catechins in the gastrointestinal tract and the permeability of the Caco-2 cell monolayer of green catechin [51]. Supercritical CO_2_ fluids can significantly affect the formation of nanoparticles by affecting the mass transfer and flow of the fluids. Among them, the nozzle structure of the supercritical CO_2_ device plays a significant role, which can affect the mixing mode of the remaining CO_2_ in the nano-solution [52]. Li et al. used supercritical CO_2_ fluid to prepare zein nanoparticles with a spherical shape, a particle size of 50–350 nm, and a filament network. It is worth noting that the morphology of zein nanoparticles is filament network, which is mainly because the large shear force generated by supercritical CO_2_ fluid can make the protein into small droplets, and promote the fusion of small protein droplets to form a filament network [53].

There are still some problems in zein-based delivery nanocarriers, including low encapsulation efficiency, poor water solubility, and poor stability [12]. These problems limit their practical application. Therefore, many scholars pay attention to the modification of zein, to improve its functional properties and expand its application in the food industry. Currently, research on the modification of zein is mainly divided into two categories: physical modification (the combination of zein and polysaccharide [75], surfactant [76], protein [77], and polyphenol [78], etc.,) and chemical modification (glycosylation [79], phosphorylation [80], deamidation[38], carboxymethylation [81], etc.,). The physical modification method can select materials with specific properties (pH response, magnetism, temperature sensitivity, etc.,) and zein solution to form a nanocomposite carrier so that the nanocarriers can quickly release active substances under specific pH, external magnetic field, and different temperature responses [82]. In addition, chemical modification can obtain some additional properties by changing the chemical groups of zein, mainly including liver protection activity, pH responsiveness, and targeting, among others [83].

### 3.2. Legume Protein-Based Nanocarriers

Soy protein is a cheap and abundant source of plant protein, which is one of the most common proteins used to manufacture food-grade nanomaterials [84]. According to its sedimentation coefficient, soy protein can be divided into four components, including 2S, 7S, 11S, and 15S. In which, 7S (β-soybean globulin) and 11S (soybean globulin) are the two main forms of globulin [85]. Soy protein-based nanocarriers mainly include nanoparticles, nanofiber aggregates, nanohydrogel, and nanotubes, among others. Some studies had reported that soy protein nanoparticles were used to load curcumin, vitamins, and β-carotene, among others.

Zhu et al. used heat-treated soybean protein isolate and epigallocatechin-3-gallate (HSPI-E) complex particles as nanocarrier, which could significantly improve the loading rate and stability of curcumin [86]. In addition, HSPI-E nanoparticles might provide a new-type carrier for medical materials. Zhang et al. prepared soy protein isolate (SPI) nanoparticles loaded with VB_12_ by using the cold gel method, and studied the mechanism of intestinal absorption and transport of SPI nanoparticles delivering VB_12_ [87]. The results showed that SPI nanoparticles crossed the small intestinal epithelial cells mainly through clathrin-mediated endocytosis and macro-pinocytosis. β-carotene is one of the common pigments with good nutrition and bioactivity. However, the low solubility and poor stability of β-carotene limited its application in the food industry [88]. SPI nanoparticles showed good enhancement capacity for water dispersibility, stability, and bioavailability of β-carotene [89].

Similar to the protein nanoparticles, protein nanofibers are widely used in nutrition and drug delivery systems because of their good biocompatibility, high emulsifying ability, and strong heat resistance [90]. Betaine has the activities of regulating the osmotic pressure in the body, increasing the activity of cellular Na/K-ATPase, promoting fat metabolism, and inhibiting fatty liver [91]. However, the low stability of betaines to thermal degradation limits their application in food processing. The results of Zhao et al. showed that soybean protein isolate nanofibers could increase the thermal stability of betaine and improve the color retention of betaine from 55.3% to 75.9% [92]. Ansarifar et al. used soy protein isolate nanofibers and high methoxy pectin to make multilayer capsules to encapsulate limonene. The results showed that the soy protein isolate nanofibers and high methoxy pectin nanocarriers had higher anti-sedimentation and flocculation stability than whey protein-encapsulated limonene [93]. Nanohydrogels are polymers with a three-dimensional network structure formed by physical or chemical cross-linking, with a high swelling ratio, water retention and gelling properties [94]. Some researchers had also reported soy-based nanogels to deliver active substances [60,61,95]. Soy protein nanoparticles, nanofibers and nanogels were used to load nutrients and active ingredients, which could significantly enhance the application value of soy protein and contribute to the construction of new-type functional foods.

### 3.3. Gliadin and Lectin-Based Nanocarriers

Gliadin (Average molecular weight: 25–100 kDa) is mainly divided into four components, named α (25–35 kDa), β (30–35 kDa), γ (35–40 kDa), and ω (55 kDa–70 kDa) [96]. Gliadin is rich in glutamine (about 40%) and proline (14%), which makes it possess amphiphilic characteristic. In addition, gliadin is also rich in lipophilic and neutral amino acids, which facilitates protein adsorption to mucosal and lipid tissue surfaces through hydrogen bonding and water transport interactions [97]. The good adhesion properties of gliadin facilitate its application in the oral delivery of hydrophobic or amphiphilic active substances and drugs. Several studies had shown that gliadin nanoparticles could effectively improve the release capacity and bioavailability of vitamin A, vitamin E, and amoxicillin, among others [98,99]. Moreover, Sonekar et al. used the desolvation method to prepare folic acid–curcumin–gliadin nanoparticles, and the results showed that the delivery vehicle could enhance the oral delivery efficiency and target colon cancer cells capacity of curcumin [100]. Furthermore, the combination of anionic polysaccharides and gliadin could prevent the aggregation of gliadin nanoparticles and improve their stability [101]. Lectin is widely used in drug delivery because it can significantly improve drug absorption and enhance sugar-targeting ability. In general, different types of cells can express different glycans, which are the main binding sites of lectin [96]. Thus, lectin can be used as carrier molecules to specifically target different cells and tissues. In addition, lectin also has strong biological adhesion, which mainly acts on the gastrointestinal tract, lung, nasal mucosa, blood-brain barrier, and eyes [97]. Interestingly, wheat lectin is the most widely used plant lectin. Gao et al. reported that wheat lectin can promote the cellular uptake of drugs by binding to receptors indicated by cells [98]. In addition, lectin-coated nanoparticles could stimulate the endocytosis of intestinal cells to transport nanoparticles out of cells, thus entering the blood circulation [99]. Moreover, lectin could combine with solid liposomes [100], proteins [101], and poly (lactic acid glycolic acid) copolymers [102] to form various types of nano carriers.

### 3.4. Other Protein-Based Nanocarriers

In addition to zein and legume protein, which are commonly used for the preparation of nanoparticles, other proteins are also used to deliver astaxanthin, curcumin, resveratrol, VD, VE, and beta-carotene, these proteins mainly include potato protein [15], rice bran protein [51], prosomillet protein [52], pea protein [14,58], gliadin protein [63], barley protein [66,67], among others. Some researches mostly focus on the entrapment, protection, and controlled release of a single bioactive substance by using plant protein as a nanocarrier. With the continuous reports of synergistic effects between a variety of bioactive substances and the increasing demand for functional foods with a variety of health benefits, the inclusion of a variety of bioactive substances in nanocarriers will also become the trend of research and development of plant protein-based nanocarriers in the future. Stratulat et al. reported that vitamin E, vitamin A, and coenzyme Q10 were co-encapsulated in cheese with good biocompatibility, which could effectively inhibit lipid oxidation [103]. In addition, zein-hyaluronic acid nanoparticles could simultaneously load curcumin (entrapment efficiency: 69.8%; loading rate: 2.5%) and quercetin (entrapment efficiency: 90.3%; loading rate: 3.5%), which could significantly improve the physical stability and gastrointestinal degradation stability of curcumin and quercetin [104]. Moreover, bovine serum albumin could bind resveratrol, retinol, and (−)-epigallocatechin-3-gallate (EGCG) because it has multiple binding sites. Compared with free resveratrol, retinol, and EGCG, bovine serum albumin tri-ligand (resveratrol, retinol and EGCG) complexes had the higher stability [105]. Therefore, it is of great significance to study the synergistic or antagonistic effects of different bioactive substances in protein carrier systems for the development of functional products.

## 4. Conclusions and Future Trends

In conclusion, plant protein-based nanocarriers show great development potential to provide controlled release of bioactive substances. Plant protein-based nanocarriers have the advantages of high nutritional value, high stability, rich resources, regeneration, and degradation. As mentioned above, plant proteins are used to prepare various nanocarriers, mainly including nanoparticles, nanofibers, nano hydrogels, etc. These nanocarriers show excellent potential for delivering bioactive substances. Although different plant-based protein nanocarriers have been developed for the protection and delivery of active substances to increase their bioavailability, there are still some issues that need to be addressed. Plant protein-based nanocarriers have been less studied in vivo, and the safety and bioavailability of nanocarriers should be studied more deeply in vivo. In addition, plant protein-based nanocarriers need to be designed to meet special delivery needs for bioactive substances with different solubility properties. Moreover, to ensure high stability, high activity retention, and bioavailability of bioactive substances, the structure–activity relationship between plant protein nanocarriers and bioactive substances needs to be further studied. This review is expected to provide theoretical support for the development of new types of plant protein nanocarriers and lay a foundation for the development of functional foods.

## Figures and Tables

**Table 1:** Comparison of characteristics between animal and plant protein-based nanocarriers.

Source	Nutrition	Environment Protection	Cost	Targeting	Biocompatibility	Drug Release Capacity
Animal protein	High	Non-renewable	High	Medium	High	Discontinuity
Plant protein	Medium	Renewable	Low	High	High	Good

**Table 2:** Summary of plant protein-based nanocarriers used to deliver active substances.

	Bioactive Substance	Wall Material	Nanoencapsulation Technique	Nanoencapsulation Type	Size (nm)	Purpose	References
Polyphenol	Resveratrol	SPI	Rotary evaporation	Nanocomplex	100	Increase solubility and drug release	[54]
	Quercetin	Zein	Electrospun	Nanofibrous	750 ± 310	Increase solubility and stability	[37]
	Quercetin	Zein	co-precipitate	Colloidal nano complex	130–161	Increase stability and antioxidant properties	[55]
	Astaxanthin	Potato protein	Freeze-drying and reconstitution	Nanoparticles	Not studied	Improve solubility and bioavailability	[15]
	EGCG	Zein	Antisolvent precipitation	Colloidal particles	170–250	Improve EE and bioactivities	[49]
	Tangeretin	Zein	Liquid–liquid dispersion	Nanoparticles	249 ± 4	Increase stability	[48]
	Cranberry procyanidins	Zein	Liquid–liquid dispersion	Nanoparticles	392–447	Increase LE	[35]
	Curcumin	SPI	Freeze-drying	Nanocomplex	Not found	Increase solubility, storage stability, bioaccessibility and digestibility	[56]
	Curcumin	Zein	Antisolvent precipitation	Nanoparticles	92.44 ± 2.24	Improve EE and stability	[50]
	Curcumin	Rice bran albumin	Magnetic stirring	Nanoparticles	120	Improve bioactivity and bioavailability	[57]
	Curcumin	Proso millet protein	Rotary evaporation	Nanoparticles	250–350	Increase solubility and heat stability	[58]
	Curcumin	*Lepidium sativum* protein hydrolysate	Freeze-drying	Nanocomplex	130–220	Increase solubility, stability, functional properties and bioaccessibility	[59]
	Curcumin	SPI	High pressure homogenization	Nanocomplex	Not studied	Increase solubility, stability and antioxidant properties	[60]
	Curcumin	Soy protein	Desolvation and rotary evaporation	Nanoparticles	220.1–286.7	Improve stability, EE and LE	[61]
	Resveratrol	Pea protein	Ca^2+^ ions induced cross-linking/cold gelation protocol	Nanoparticles	207.6	Improve EE and LE	[14]
Vitamins	Vitamin D3	Corn protein hydrolysate	Freeze-drying	Nanocomplex	102–121	Increase stability and bioaccessibility	[62]
	Vitamin D3	Soy protein	Ionic gelation	Nanoparticles	162–243	Improve EE and LE	[63]
	Vitamin D	Pea protein	High pressure homogenization	Nanoemulsions	170–350	Improve EE and bioavailability	[64]
	Riboflavin	Soy protein	Salt-induced gelation	Nano-hydrogels	Not studied	Increase bioaccessibility	[65]
	Riboflavin	Soy protein	Ultrasound pre-treatment and transglutaminase-induced gels	Nano-hydrogels	Not studied	Increase gel strength, EE and gel yield; Decrease the digestibility	[66]
	Folic acid	Soy protein	High pressure homogenization and heat	Nanogels	Not studied	Increase EE and stability	[67]
	Vitamin D3	Zein	Phase separation (liquid-liquid dispersion) and freeze-drying	Nanoparticles	86–200	Increase EE and stability	[47]
	Vitamin E	Gliadin	Desolvation	Nanoparticles	450–475	Increase EE	[68]
Others	RA	Gliadin	Desolvation	Nanoparticles	500	Increase EE and controlled release	[69]
	Cyclophosphamide	Gliadin	Electrospray deposition	Nanoparticles	Not studied	Increase controlled release	[70]
	β- carotene	Barley protein	Spray-dried and enzymatic degradation	Nano-encapsulations	20–30	Increase controlled release	[71]
	β- carotene	Barley protein	High pressure homogenization	Nanoparticles	90–150	Increase storage stability and bioaccessibility	[72]
	Essential oils	Zein	High-speed mix	Nanospherical particles	Not found	Increase bioaccessibility	[73]
	Essential oils	Zein	Liquid–liquid dispersion	Nanoparticles	Not found	Increase solubility, antimicrobial and antioxidant properties	[33]
	Lutein	Zein	SEDS	Nanoparticles	Not found	Increase EE and controlled release	[74]

Note: EGCG represents epigallocatechin gallate; EE represents encapsulation efficiency; SPI represents soy protein isolate; LE represents loading efficiency; RA represents all-trans-retinoic acid; SEDS represents solution enhanced dispersion by supercritical fluids.

## Data Availability

Not applicable.
